# Artificial intelligence for tuberculosis control: a scoping review of applications in public health

**DOI:** 10.7189/jogh.15.04192

**Published:** 2025-07-25

**Authors:** Sonia Menon, Kobto Ghislain Koura

**Affiliations:** 1 International Union against Tuberculosis and Lung Disease, Paris, France; 2 Epitech Research, Auderghem, Belgium; 3 UMR261 MERIT, Université Paris Cité, IRD, Paris, France

## Abstract

**Background:**

Artificial intelligence (AI) has become an important tool in global health, improving disease diagnosis and management. Despite advancements, tuberculosis (TB) remains a public health challenge, particularly in low- and middle-income countries where diagnostic methods are limited. In this scoping review, we aim to examine the potential role of AI in TB control.

**Methods:**

We conducted a search on 25 August 2024 for the past five years, in the PubMed database using keywords related to AI and TB. We included laboratory-based and observational studies focussing on AI applications in TB, excluding non-original research.

**Results:**

There were 34 eligible studies, identifying eight overarching aspects associated with TB control, including active case finding (ACF), triage, pleural effusion diagnosis, multidrug-resistant (MDR) TB and extensively drug-resistant (XDR) TB, differential diagnosis distinguishing active TB from TB infection and other pulmonary communicable diseases, TB and other pulmonary communicable and non-communicable diseases (NCDs), treatment outcome prediction, pleural effusion, and predictions of regional and national trends. AI may transform TB control through enhanced ACF methods and triage, improving detection rates in high-burden regions. With high accuracy, AI may diagnose pleural diagnosis, differentiate TB active and TB infection, TB and non-tuberculous mycobacterial lung disease, COVID-19, and pulmonary NCDs. AI applications may facilitate the prediction of treatment success and adverse effects. Furthermore, AI-driven hotspot mapping may identify undiagnosed TB cases at rates surpassing traditional notification methods. Lastly, predictive modelling and clinical decision support systems may improve the management of MDR-TB.

**Conclusions:**

This scoping review highlights the potential of AI-driven predictions in national TB programmes to enhance diagnostics, track trends, and strengthen public health surveillance. While promising for reducing transmission and supporting TB care in low-resource settings, these models require large-scale validation to ensure real-world applicability, especially for high-risk groups.

Tuberculosis (TB) remains a critical global health issue, with the World Health Organization (WHO) ranking it the second cause of death from a single infectious agent, after COVID-19 [1]. Despite progress in TB diagnosis, treatment, and prevention, the disease continues to pose significant public health challenges due to the emergence of drug-resistant strains [2] and its strong association with social determinants of health [3]. TB is particularly pronounced in low- and middle-income countries (LMICs), where the burden of the disease is highest, co-existing with other lower respiratory tract infections [4] and pulmonary non-communicable diseases (NCDs) [5], with limited health care resources.

The diagnostic landscape for TB remains challenging. No single test reliably distinguishes between active and latent TB. Tests like the tuberculin skin test and interferon-gamma release assays have lower performance in detecting TB in countries with high TB incidence [6]. About 50% of individuals previously exposed to *M. tuberculosis* may not react to the tuberculin skin test due to impaired type 1 immunity or lack of infection [7]. Whilst culture remains the gold standard for TB diagnosis, it is time-consuming [8] and have limited sensitivity [9]. Molecular tests like GeneXpert and TrueNAT have improved accuracy, but are expensive [10,11]. Affordable alternatives such as chest x-rays and computed tomography (CT) scans are crucial, while more sensitive in detecting microscopic lung lesions, depend heavily on the experience of the physician [12]. However, the shortage of radiology specialists and the variability in TB CT features further complicate accurate diagnosis, making it a time-consuming and challenging process.

Against this backdrop, artificial intelligence (AI) has been gaining traction through machine learning (ML), deep learning and natural language processing [13], enhancing disease surveillance [14], diagnostic accuracy [15], treatment protocols [16], and outbreak patterns predictions [17]. AI is already deployed in medical imaging [18], with evidence demonstrating that AI-based software could serve as an accurate tool to diagnose pulmonary TB in medical imaging [19] and that deep learning (DL) holds promise for improving smear microscopy [20].

Despite growing interest in integrating AI in TB control, no comprehensive synthesis currently examines the scope of AI applications across the TB care cascade and their broader public health implications. While AI holds promise in addressing persistent challenges in TB diagnosis, treatment monitoring, and surveillance, particularly in resource-limited settings, the evidence remains fragmented. We aim to systematically identify and categorise recent applications of AI in TB control, highlight current limitations, and uncover critical research gaps. By focussing on studies published over the past five years, we aim to provide an up-to-date foundation to inform future research, support evidence-based implementation, and guide subsequent systematic reviews.

## METHODS

Given the methodological heterogeneity and early research stage in this field, we chose a scoping review to map the landscape of AI applications in TB control and identify research gaps. This approach was better suited to synthesising the breadth of evidence in a rapidly evolving field. A systematic review at this stage would be premature due to the lack of up-to-date and robust evaluation frameworks [21] and the rapid evolution of AI methodologies, making quality assessment challenging. While a scoping review does not typically include a formal quality assessment [22], it provides an overview of the available evidence to determine whether a full systematic review would be feasible or beneficial.

We conducted a literature search in PubMed on 25 August 2024, using AI- and TB-related keywords, with no language or publication date restrictions. We screened reference lists of relevant articles for additional studies to ensure a thorough review. We registered the protocol with OSF [23]. Our primary research question guiding this scoping review was: ‘In what areas of TB control has AI been applied?’

To structure the review, we used the PICO framework:

- Population: Individuals diagnosed with TB, TB-affected populations, or those at risk.
- Intervention: AI applications in TB diagnostics, treatment, or epidemiological control.
- Comparison: Traditional (non-AI) epidemiological or diagnostic methods.
- Outcome: Mapping AI’s contributions to TB diagnosis, treatment, and epidemiology over the past five years.

### Inclusion and exclusion criteria

Peer-reviewed articles included in this review were laboratory-based studies and observational studies focussing on the application of AI in TB, irrespective of the language. We excluded non-original peer-reviewed research such as commentaries, editorials, and review articles.

### Data extraction, synthesis, and reporting

We extracted the data on the types of AI technologies employed in the studies. Whenever diagnostic performance was assessed using standard metrics, such as sensitivity and/or specificity, the area under the receiver operating characteristic curve (AUC-ROC) was reported to compare the discriminative ability of different models, with higher values indicating better discrimination between patients who are high-risk and low-risk [24].

We synthesised the key findings into categories that emerged organically from the literature, focussing on AI's role in TB control and comparing outcomes between traditional and AI-driven approaches. We followed the PRISMA-ScR guidelines in reporting our findings [25] (Checklist S1 in the **Online Supplementary Document**).

## RESULTS

**Figure 1:**
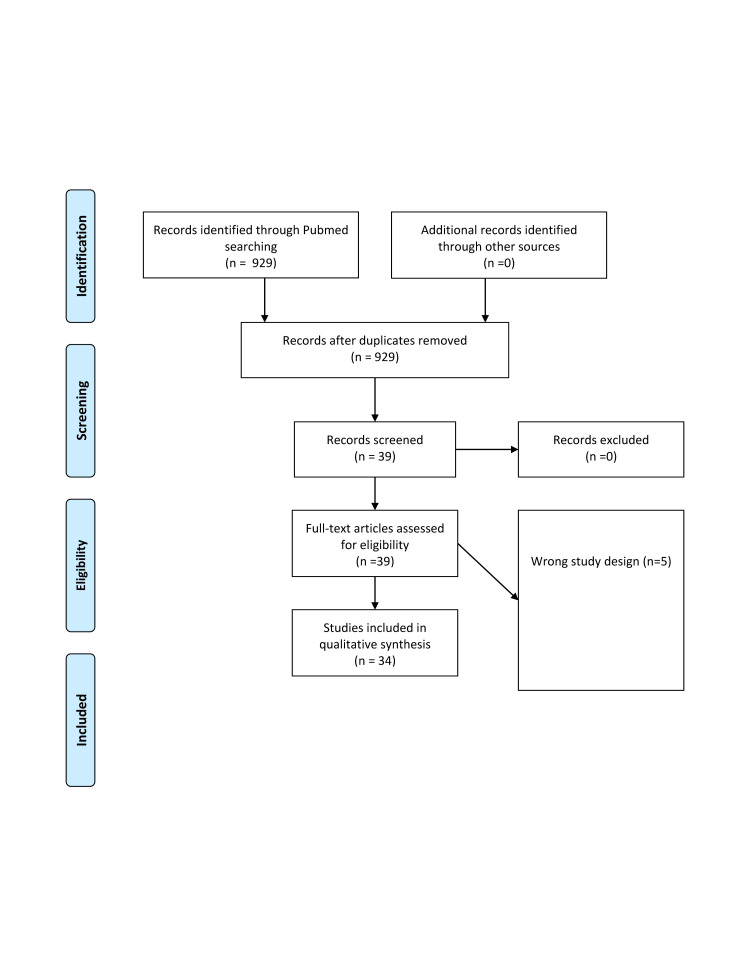
PRISMA flowchart. Adapted from [26].

Using the broadest search terms in our PubMed search and from a search through the references of eligible papers, 929 studies were identified. Of these, 895 were excluded as the study design was not eligible, leaving us with 34 studies (**Figure 1**). From the 34 studies eligible, we identified eight overarching aspects associated with TB control, active case finding (ACF), triage, pleural effusion diagnosis, Multidrug-resistant (MDR) TB and extensive drug-resistant (XDR) TB, differential diagnosis with other pulmonary communicable and NCDs, treatment outcomes predictions, predictions or regional or national trends (Table S1 in the **Online Supplementary Document**).

### ACF

Two multicentric studies have highlighted the integration of advanced methodologies in ACF for TB, emphasising their potential to enhance detection rates in high-burden areas. Okada et al. [27] assessed AI-based computer-aided detection (CAD) systems in a community-based ACF initiative in Cambodia, targeting individuals aged ≥55, symptomatic patients, and those at elevated risk. Using Xpert-positive TB or human interpretation as a reference, the study demonstrated that AI-CAD scores were significantly correlated with chest radiography classifications, achieving an AUC-ROC of 0.86 (95% confidence interval (CI) = 0.83–0.89). The AI-CAD system reduced the workload for human CXR readers by 21% and for bacteriological testing by 15%, while still detecting 95% of the TB cases confirmed as positive by the Xpert test. Also, in Nigeria, Alege et al. [28] showed how AI-driven hotspot mapping for ACF outperforms traditional notification data in identifying undiagnosed TB hotspots. Analysing data from an ACF intervention across four southwestern states, researchers used a Bayesian inference model to predict TB positivity rates, revealing that AI-identified hotspots had at least 1.75 times higher rates (*P* < 0.001) than those detected through conventional methods.

### Triage

WHO has identified the use of triage tests as one potential solution for improving TB diagnostic pathways in resource-limited setting [29]. AI and ML are making headway in TB triage, with four key studies demonstrating the potential of AI-driven triage to enhance TB detection and management.

Khan et al. [30] evaluated qXR, version 2.0, and CAD4TB, version 6.0, against mycobacterial culture in 2370 symptomatic adults in Karachi, Pakistan, where 99.5% of participants were HIV-negative. Both AI tools met WHO's TB triage criteria with 0.93 sensitivity (95% CI = 0.89–0.95) and (95% CI = 0.90–0.96), respectively. Specificity was 0.75(95% CI = 0.73–0.77) for qXR and 0.69 (95% CI = 0.67–0.71) for CAD4TB, highlighting AI’s potential in radiographic interpretation where experts are scarce. Also in the same city, Nsengiyumva et al. (2021) [31] evaluated AI-based CXR triage in a TB clinic, using an HIV-negative cohort with TB symptoms. AI successfully identified individuals needing further microbiological testing, reducing costs by 19–37% and averting 3–4% more disability-adjusted life years compared to smear microscopy or GeneXpert. Similarly, Bosman et al. (2024) [32] evaluated CAD4TBv7 for TB detection compared to a C-reactive protein assay for TB triage in 1392 symptomatic adults in Lesotho and South Africa, where 48% of participants were HIV-positive. CAD4TBv7 achieved an AUC of 0.87 (95% CI = 0.84–0.91) with 68.2% (95% CI = 65.4–71.0%) specificity at 90% sensitivity, comparable to an expert radiologist and approaching WHO’s target product profile, while C-reactive protein showed a much lower specificity (38.2%; 95% CI = 35.3–41.1%). Finally, Biewer et al. (2024) [33] evaluated the qXR software in a Peruvian hospital as part of the FAST strategy (*i.e.* find cases actively, separate safely, and treat effectively). Among 1006 patients enrolled and admitted to the tertiary hospital in Lima, Peru, qXR demonstrated high sensitivity 0.91 (95% CI = 0.81–0.97) using culture as the reference standard and 0.93 (95% CI = 0.84–0.98) compared to Xpert. However, its specificity was low, at 0.32 (95% CI = 0.27–0.37) for triaging patients with cough or TB risk factors, using culture as the reference standard compared to culture and Xpert, respectively.

### Pleural effusion diagnosis

Early and accurate diagnosis of tuberculous pleural effusion (TPE) is crucial for improving patient outcomes and controlling TB, and two single-centred studies examine its application in this field. In China, Liu et al. [34] developed nine ML models to diagnose TPE in 1435 untreated patients. The support vector machine performed best, achieving 87.7% balanced accuracy, 85.3% precision, and an AUC of 0.914. It demonstrated high sensitivity (94.7%) and specificity (80.7%), with external validation confirming its potential robustness for clinical use. Also, Ren et al. [35] analysed data from 192 TPE patients, of which 54 had parapneumonic pleural effusion and 197 had malignant pleural effusion, employing four ML algorithms. The random forest (RF) model outperformed the others, achieving sensitivity and specificity rates of 89.1% and 93.6%, respectively, surpassing conventional testing using pleural fluid adenosine deaminase’s sensitivity of 85.4% and specificity of 84.1%. A refined RF model, leveraging 12 key features, further improved performance, achieving 100% sensitivity and 90% specificity.

### MDR-TB and XDR-TB

AI has significantly advanced MDR-TB and XDR-TB research, particularly in diagnostics, predictive modelling, personalised medicine, and transmission analysis. Eight studies explore these advancements.

In Thailand, the TB-DRD-CXR web application was assessed by Sethanan et al. [36] employing ensemble deep learning models to classify TB patients, achieved an accuracy of 96.7%, improved diagnostic precision by 4.0–33.9%, outperforming standard convolutional neural network architectures. The TB-DRD-CXR web application, tested by 33 medical staff, demonstrated 96.7% accuracy, an efficiency rate of 4.16 goals/min, and an overall relative efficiency of 100%. With a system usability scale score of 96.7%, the application shows high user satisfaction and strong adoption potential. Similarly, Herman et al. [37] assessed the artificial neural networks-based CUHAS-ROBUST model to detect pulmonary rifampicin-resistant TB in a study of 487 participants in Indonesia. It outperformed other AI classifiers, achieving the highest accuracy (88%; 95% CI = 85–91) and sensitivity (84%; 95% CI = 76–89), though with lower specificity (90%; 95% CI = 86–93) compared to logistic regression (99%; 95% CI = 97–99), suggesting it may enhance rifampicin-resistant screening, particularly in settings without GeneXpert access.

In addition, using the public database (TB DEPOT), Tulo et al. [38] demonstrated that segmentation methods effectively delineate the lungs and mediastinum on CXR images, with extracted features significantly differentiating drug-sensitive (DS) and drug-resistant (DR) TB (*P* < 0.05). Using both feature sets, a multi-layer perceptron classifier achieved F-measures of 82.4% (DS *vs* MDR), 81.0% (MDR *vs* XDR), and 87.0% (DS *vs* XDR), suggesting that incorporating mediastinal analysis alongside lung features may enhance the diagnostic performance for distinguishing drug-sensitive and drug-resistant TB.

In genomic resistance prediction, compared to GeneXpert-MTB/RIF, Portelli et al. [39] illustrated how SUSPECT-RIF (StrUctural Susceptibility Prediction for Rifampin) may accurately identify rpoB gene mutations associated with rifampicin resistance, achieving a sensitivity of 92.2% and a specificity of 83.6%. Verboven et al. [40] compared an expert recommendation to an automated clinical decision support system, which recommended treatments based on patient profiles, attaining a precision rate of 95% in identifying appropriate regimens for drug-resistant TB in 355 patients. Li et al. [41] developed a model to predict MDR cavitary pulmonary tuberculosis using CT radiomics features in China. The study involved 257 patients, 187 in the training and 70 in the testing cohorts. The radiomics model outperformed the clinical model, with AUCs of 0.844 *vs* 0.589 (training) and 0.829 *vs* 0.500 (testing), both *P* < 0.05. The combined model slightly outperformed the radiomics model, but the difference was insignificant. Additionally, Yang et al. [42] developed ML models using DNA sequencing data from 1839 isolates (UK, Sierra Leone, South Africa, Germany, and Uzbekistan) to classify resistance against eight anti-TB drugs, achieving higher sensitivities ranging from 84% to 97% for various drugs, with improvements of up to 24% for pyrazinamide and streptomycin, compared to traditional molecular diagnostic methods. Lastly, Sibandze et al. [43] in South Africa employed AI to investigate drug resistance patterns in extrapulmonary TB, analysing 70 culture-positive non-pulmonary samples revealed that 36% of strains belonged to the East Asian lineage, which was significantly associated with drug resistance (odds ratio (OR) = 12.69; 95% CI = 1.82–141.60), although the study did not use a control group.

### Differential diagnosis distinguishing active TB from TB infection and other pulmonary communicable diseases

Six multicentric studies highlighted AI's potential to enhance the differentiation between active TB and TB infection and distinguish TB from nontuberculous mycobacterial lung diseases (NTM-LDs).

Luo et al. [44] developed 28 ML models that use multiple laboratory data, including T-SPOT assays and lymphocyte characteristics, to distinguish active TB from TB infection. In a study of 892 participants from central China's largest hospital, the conditional forest model showed that the ROC showed limited value of individual laboratory indicators for differentiating active TB from TB infection (<0.8). In contrast, the conditional forest model presented an AUC of 0.978, with a sensitivity of 93.39% and a specificity of 91.18%, when using culture or GeneXpert MTB/RIF.

Similarly, Nijiati et al. [45], in a retrospective analysis of 2291 hospital patients in China, including 1160 patients with active TB and 1131 patients with non-active TB cases, advanced deep learning models were employed to analyse CT scans and clinical data. The 3D ResNet-50 model achieved an AUC of 0.96, outperforming experienced radiologists.

AI's application extends to differentiating pulmonary TB from NTM-LD, Liu et al. [46] analysed 1500 chest x-rays from two hospitals using a deep neural network. The model achieved AUCs of 0.83 and 0.76 for TB, 0.86 and 0.64 for NTM-LD, outperforming experienced pulmonologists, indicating its potential as a reliable first-line screening tool. Building on AI’s diagnostic potential, Wang et al. [47] developed a 3D-ResNet deep learning framework to distinguish NTM-LD from *M.tuberculosis* lung disease, using chest CT images of 301 with NTM lung disease and 804 with mycobacterium TB-LDs collected in China. The model yielded an AUCs of 0.90 during training and 0.86 during testing, surpassing radiologists in accuracy and demonstrating AI’s ability to identify lung abnormalities rapidly.

In the context of COVID-19 in China, Yang et al. [42] developed a ResUNet-based deep learning algorithm to enhance the differentiation of COVID-19 pneumonia from other pulmonary infections on CT scans. This model achieved 91.4% accuracy and an AUC of 0.903, demonstrating that AI can significantly improve diagnostic performance for various respiratory diseases in the 185 chest CT images, compared to without its assistance. Furthermore, Nabulsi et al. [48] developed an AI system to classify chest x-rays, reducing diagnosis turnaround time and detecting previously unseen diseases. Trained on 248 445 patients from a multi-city hospital network in India, the system was tested on six data sets, achieving AUCs varying between 0.95 (95% CI = 0.93–0.97) and 0.97 (95% CI = 0.94–0.99) for both TB data sets.

### Differential diagnosis distinguishing active tuberculosis from pulmonary NCDs

There are complex interactions between TB and various pulmonary NCDs [49], which are often diagnosed concurrently. The presence of one disease can increase the risk of developing the other [50]. Two studies explore the use of AI to differentiate TB from silicosis, a lung disease caused by inhaling large amounts of crystalline silica dust, which increases the risk of TB [51] and from lung adenocarcinoma.

In China, Liu et al. [52] developed a radiomics-based prediction model using CT images to differentiate between silicosis and TB nodules in a hospital setting. The study analysed 139 silicosis and 119 TB lesions. The RF model achieved 83.1% accuracy, 76% sensitivity, 90% specificity, and an AUC of 0.917 (95% CI = 0.84-0.97), outperforming support vector machine and feedforward neural network models (*P* < 0.05), suggesting that the RF model is effective for differentiating silicosis from TB nodules. Furthermore, Feng et al. [53] developed a CT-based deep learning nomogram to differentiate tuberculous granulomas from lung adenocarcinomas in solitary solid pulmonary nodules. Using retrospective data from 550 patients, a convolutional neural network extracted deep learning features. Independent predictors, including deep learning signature, age, gender, and lobulated shape, were integrated into the deep learning nomogram, which demonstrated superior diagnostic accuracy with AUCs of 0.81 (95% CI = 0.75–0.86) in the external validation cohorts.

### Treatment outcomes prediction

AI and ML approaches are increasingly being used to predict and improve treatment outcomes in pulmonary TB. Five multicentric studies, of which two did not compare with any traditional statistical methods, illustrate this.

Kim et al. [54] explored factors influencing treatment success and culture conversion using AI-based chest x-ray analysis and GeneXpert MTB/RIF assay cycle threshold values across six South Korean centres. In this study of 230 patients with rifampicin-susceptible TB, AI-based radiographic scores emerged as significant predictors of both treatment success (OR = 0.94; 95% CI = 0.91–0.97) and culture conversion at eight weeks (OR = 0.91; 95% CI = 0.85–0.97). Xpert cycle threshold values, however, did not show a significant correlation with outcomes. Similarly, in Japan, Higashiguchi et al. [55] utilised convolutional neural networks and chest radiography to predict the time to culture negativity in active pulmonary TB using chest radiographs in 239 hospital-admitted patients, randomised to either the training or the validation data set group. Although the convolutional neural network model demonstrated only moderate accuracy (Pearson correlation coefficient = 0.392; *P* = 0.002), the model’s predicted timing for culture conversion was, on average, either 18 days earlier or later.

Addressing the challenge of treatment failure, Asad et al. [56] applied ML and data analytics to identify key predictors of failure in TB treatment using data from high-burden countries. The study demonstrated the potential of these predictors to improve patient outcomes, achieving 78% accuracy overall and 92% accuracy in the Romanian cohort, underscoring the importance of tailored interventions in high-risk populations. Rodrigues et al. [57] developed a risk score to predict loss to follow-up during TB treatment using the Brazilian Notifiable Disease Information System data for 2015–22. This retrospective study analysed 243 726 cases, excluding individuals under 18 and those with drug-resistant TB. Among these, 41 373 experienced loss to follow-up, and its risk was calculated using key features including prior TB, drug use, age, sex, HIV status, and education level, with models achieving an AUC of 0.71–0.72. Lastly, Liao et al. [58] focussed on predicting adverse effects such as acute hepatitis, respiratory failure, and mortality in TB patients using AI models. With data from 2248 patients in a hospital in Taiwan, the study compared the performance of AI models, including XGBoost, Random Forest, MLP, LightGBM, and support vector machine, against traditional statistical methods, specifically Logistic Regression. The AI models demonstrated high predictive accuracy across these outcomes, with AUCs ranging from 0.920 to 0.766 for hepatitis, 0.884 to 0.797 for respiratory failure, and 0.834 to 0.737 for mortality.

### Predictions of regional or national trends

Accurate forecasting of TB trends is crucial for improving prevention, control strategies, and resource allocation. Five studies have demonstrated the potential of ML and AI in enhancing predictions by using a combination of epidemiological, sociodemographic, and environmental data.

Modidem et al. [59] demonstrated the superiority of artificial neural networks over traditional multiple linear regression models in predicting TB cases in Malaysia, achieving over 96% accuracy. The best model, ANN3, incorporated sociodemographic factors (*e.g.* nationality, residency, income) and environmental variables, resulting in an R^2^ of 0.47, errors <6, and accuracy exceeding 96%. The study suggests that artificial neural network models leveraging sociodemographic and environmental data offer more accurate predictions of TB cases. Dixit et al. [60] used pathogen genomics and a validated RF model to predict resistance to 12 drugs, analysing global *M.tuberculosis* surveillance data from 29 countries. Their study revealed high levels of levofloxacin resistance in South Asia, highlighting concerns over widespread fluoroquinolone use, while also suggesting underutilisation of ethionamide for treating isoniazid-resistant TB in MDR cases. Validation against South Africa's national drug resistance survey showed strong alignment, except for underestimations in isoniazid, ethionamide, and second-line injectables.

In Russia, Chernianev et al. [61] used AI-based models to predict TB trends in a high-HIV-burden region. Comparing TB predictions from 2017 with data from 2018–21 showed a strong alignment in epidemiological trends, with a maximum deviation of 14.8%, underscoring its potential use in TB planning. Finally, Abade et al. [62] and Silva et al. [63], both ecological time series studies highlighted the effectiveness of deep learning and ML models in predicting TB/HIV co-infection trends in Central West Brazil and identifying TB hotspots in the Amazon, respectively, outperforming traditional statistical models.

## DISCUSSION

Our scoping review, consisting of 34 studies, shows that AI research spans across several areas of TB research, including its role in ACF, triage, pleural effusion, early prediction of MDR-TB and XDR-TB, differential diagnosis from other pulmonary communicable and NCDs, treatment outcomes predictions, and forecasting national TB trends. Our findings highlight how advancements in AI can impact public health by streamlining TB control efforts in resource-limited settings, reducing transmission, hospital stays, and health care costs.

By potentially improving detection rates in symptomatic individuals, especially in areas with limited radiological expertise, automating x-ray interpretation may enhance the efficiency of community-based ACF programmes, resulting in timelier TB diagnosis and treatment initiation in high-prevalence settings. Additionally, evidence suggests that the validation of ML diagnostic models for early diagnosis of TPE may prove particularly beneficial, especially in high HIV prevalence settings [64], further strengthening the integration of AI in TB control.

AI-powered CXR diagnosis also shows promise for mass screening and faster TB detection, reducing reliance on empirical treatment and curbing XDR-TB risk. As a corollary, early personalised MDR-TB treatment may further prevent resistance by addressing weak health care systems, improper treatment, and community-level transmission [65]. While AI-based models using chest x-ray features have shown potential for differentiating DS, MDR, and XDR TB with high accuracy, such approaches are relying on anatomical abnormalities and structural markers observed in radiographs rather than detecting specific resistance-conferring mutations. These models may thus reflect advanced disease stages or chronic pathological patterns associated with resistance. Importantly, WHO-endorsed diagnostics, such as GeneXpert MTB/RIF, line probe assays, and culture-based drug susceptibility testing, remain the standard tools for MDR-TB diagnosis. AI-based imaging models should therefore be considered complementary, not substitutive, and may be best positioned as triage tools to optimise testing efficiency, especially in settings with limited laboratory infrastructure.

Using diverse data sources, such as laboratory results and imaging studies, AI models suggest superior accuracy and efficiency compared to traditional diagnostic methods. AI's ability to distinguish between active and TB infection, a key challenge, could enhance TB management by improving diagnostic accuracy and optimising treatment allocation. For active TB, earlier detection facilitated by AI could prompt appropriate treatment initiation, thereby reducing transmission and enhancing patient outcomes. Conversely, AI could prevent unnecessary treatment in individuals with latent TB, minimising exposure to potentially adverse effects of TB regimens while conserving resources.

AI has demonstrated the potential to differentiate TB from lung diseases attributable to other pulmonary communicable and pulmonary NCDs. Historically, the focus on pulmonary TB has led to under-recognition of NTM-LD, often resulting in unnecessary empirical TB treatment. Amidst a rising global mortality rate due to NTM-LD, particularly among both immunocompetent and immunocompromised populations [66], there is emerging evidence that AI can substantially improve accuracy in diagnosing the disease, reducing misdiagnoses, and facilitating timely and appropriate treatment. Differentiating mycobacterium TB-LDs from NTM-LD remains a clinical challenge, as commonly used tests like the purified protein derivative test and AFB smears cannot distinguish between the two [67], while bacterial cultures, though definitive, are time-consuming. In TB-endemic areas, empirical anti-TB treatment for positive AFB smears delays effective NTM-LD management [68], worsening disease progression and increasing treatment failure. Furthermore, given the prolonged presence of COVID-19, AI’s ability to differentiate COVID-19 from TB could reduce testing delays, expedite treatment, transmission within communities [69], and identify co-infected individuals at greater risk for severe forms of both diseases [70].

Emerging advancements in AI may also enhance diagnostic accuracy, differentiating TB from pulmonary NCDs, including silicosis, which frequently co-occur in many working populations. This dual burden, known as silico-tuberculosis [51], is prompting renewed global attention as part of efforts to combat the TB epidemic in LMICs [71], where high TB prevalence and occupational silica exposure intersect. Additionally, higher smoking rates among TB patients may further elevate the risk of lung adenocarcinoma, highlighting AI’s role in improving early detection and patient outcomes.

Our findings highlight the economic and clinical advantages of integrating AI into TB, particularly in low-resource, high-burden settings. However, the effectiveness of these tools depends on precise calibration of sensitivity and specificity thresholds tailored to diverse clinical environments. Evidence, primarily from HIV-negative populations, demonstrates that AI-based chest x-ray tools exhibit high sensitivity and acceptable specificity, meeting WHO standards for TB triage. These technologies offer promising solutions in settings with limited radiological expertise, reducing costs and improving outcomes through more efficient patient triage. Moreover, AI-driven triage has been shown to avert disability-adjusted life years more than conventional approaches. Despite these benefits, challenges remain in generalising diagnostic accuracy across diverse populations. In cohorts with high rates of smear-negative TB, older or underweight individuals, or those with a history of TB, AI tools may not consistently achieve the sensitivity and specificity thresholds set by the WHO. This underscores the need for further optimisation and validation of AI models in heterogeneous clinical settings.

While evidence is still emerging, AI's potential to predict TB drug resistance from genomic data using ML could guide targeted public health interventions. Genomic resistance prediction models, including those analysing rpoB gene mutations, missed in approximately one-third of resistant TB infections [72] may enhance real-time surveillance and facilitate prompt public health responses, especially in resource-limited settings.

Also, evidence from this scoping review suggests that AI technologies may improve monitoring and prediction of treatment success, time to culture negativity, and patient adherence. AI may support targeted interventions that enhance patient outcomes by identifying predictors of treatment failure and loss to follow-up. Findings also suggest that a nationwide risk score has the potential to inform decision-making strategies for resource allocation and directly-observed therapy, ultimately improving TB treatment adherence. Furthermore, drawing upon laboratory data and patient characteristics, AI models may enable predictions of adverse treatment effects due to isoniazid and rifampicin, aiding early detection of hepatitis, which has been estimated at 2.55% in a meta-analysis [73]. Another promising application is AI-driven TB forecasting, where integrating epidemiological, sociodemographic, and environmental data can refine trend predictions, optimise resource distribution, and enable more targeted interventions, particularly in high-burden regions. Additionally, an AI model combining sociodemographic and environmental data predicted TB trends with over 95% accuracy. While promising, it was trained on retrospective data and lacks real-world validation.

The literature reveals a range of methodologies, including traditional statistical models, ML, and DL, each offering distinct advantages for TB control. Traditional models, such as logistic regression, are hypothesis-driven and valued for their transparency and interpretability, especially when working with structured data [74]. ML methods, including RFs, support vector machines, and gradient boosting, excel at identifying complex, nonlinear patterns, but they often come at the cost of interpretability [75]. DL, a subset of ML that uses multilayer neural networks, is particularly effective in analysing unstructured data like chest x-rays and CT scans, achieving high diagnostic accuracy [76]. However, DL models require large data sets, significant computational resources, and often operate as ‘black boxes’ due to their limited transparency [77]. These distinctions are crucial when considering implementing these technologies in real-world TB programmes, especially in low-resource settings where model interpretability, computational efficiency, and integration of existing health care infrastructure are key.

### Strengths and limitations

This scoping review is strengthened by its broad inclusion criteria, incorporating studies across languages and diverse populations, providing a global perspective on AI’s potential in TB diagnosis, management, and surveillance. However, several methodological limitations must be acknowledged. The reliance on PubMed as the primary database may have excluded relevant studies from other sources, including grey literature. Publication bias in AI research is another concern, as studies with null results are less likely to be published [78], leading to an overrepresentation of positive findings and an incomplete picture of AI’s real-world effectiveness. Furthermore, the dominance of private equity firms and big tech companies in AI research [79] may reduce the emphasis on publishing findings in academic literature [80], further skewing the available evidence. Another key limitation is using AUC as a performance metric, as a high AUC does not guarantee clinical utility [24].

While most multicentric studies were often confined to the same geographic region, this limited their broader applicability. The reliance on retrospective case analyses with convenience sampling in urban health care settings, often lacking well-defined population characteristics, may introduce selection bias and limit real-world applicability beyond specific clinical contexts. Focussing solely on adults with specific TB symptoms may further limit the applicability of findings to broader populations, including asymptomatic, less symptomatic individuals or those with more atypical presentations. Research conducted in urban tertiary health care settings, which primarily treat more severe cases, may lead to spectrum bias, as the patient population may not reflect those with milder forms of the disease seen in primary or community care. The exclusion of severely ill patients could also introduce verification bias, potentially overestimating test accuracy due to differential disease status verification. Furthermore, the lack of explicit reporting on blinding raises concerns about possible confirmation bias in AI model evaluation. While studies show promising results for AI in predicting TB treatment outcomes, the evidence is limited by the lack of comparisons with traditional prediction models, preventing definitive conclusions on AI’s superiority. Finally, the absence of prospective studies with consecutive or random sampling, especially in high-burden regions like sub-Saharan Africa, weakens external validity and calls for more geographically diverse research.

### Research gaps

This review identifies several critical epidemiological research gaps in the application of AI for TB management.

Prospective validation of AI models for predicting treatment outcomes in real-world settings is lacking. Moreover, direct comparisons between AI-based and traditional prediction models are needed to better assess whether AI consistently outperforms conventional approaches in predicting treatment outcomes.

Another significant gap lies in the lack of studies focussed on paediatric populations, where TB may present differently from adults, requiring specialised diagnostic approaches. Additionally, there is an absence of research exploring AI's ability to differentiate TB from tobacco-related diseases, such as lung cancer and chronic bronchitis, which share overlapping clinical features. This gap is particularly relevant in high TB prevalence settings with elevated smoking rates, where distinguishing between these conditions is crucial for effective diagnosis.

Moreover, there is a need for further investigation into the potential biases inherent in hospital-based participant enrolment, especially regarding individuals with TB infection. Studies indicate that in areas with high rates of smear-negative pulmonary TB, AI models may struggle to meet the WHO’s sensitivity criteria, particularly among older adults, individuals with a history of pulmonary TB, those with underweight status, or patients with small pulmonary lesions. Therefore, rigorous validation of AI models is essential to assess their efficacy across diverse, high-risk populations and settings, including primary care environments.

One study in this review developed AI-based prediction models for TB trends using sociodemographic and environmental variables. The best-performing model, a neural network combining both data types, reported a high accuracy above 95%. While the results are encouraging, the model was based on retrospective data and has not yet been validated in real-world settings. Prospective validation and implementation studies are needed to assess feasibility and effectiveness in routine TB surveillance systems.

Furthermore, while AI has shown promise in TB diagnostics, there is a lack of studies examining its role in detecting asymptomatic TB, which may contribute to TB transmission. Investigating AI’s potential to detect asymptomatic TB could significantly improve early detection strategies and reduce transmission. Additionally, no studies have explored AI's capacity to predict long-term complications in TB survivors. Addressing this gap could substantially improve post-TB care and reduce the burden of chronic sequelae, contributing to better patient management and public health outcomes.

AI applications in TB management remain underexplored in underserved populations, such as migrant workers, refugees, and incarcerated individuals, where health care access is limited. Existing models are trained mainly on data from urban or well-resourced settings, raising concerns about their real-world applicability. Without validation in diverse, high-risk groups, AI risks reinforcing health disparities rather than bridging diagnostic gaps. Additionally, socioeconomic factors, such as access to health care, education, and computational resources, must be considered in understanding how AI technologies can be applied in real-world settings. The interaction between social determinants of health and AI-driven interventions may significantly influence the effectiveness and equity of AI solutions in TB management.

AI’s role in detecting asymptomatic TB, a potential driver of transmission, remains largely unexplored, as does its capacity to predict long-term complications in TB survivors. Addressing these gaps could enhance early detection strategies and post-TB care, reduce chronic sequelae and improve patient outcomes. Furthermore, current AI applications in TB predominantly focus on high-income settings, often neglecting the populations most affected in LMICs. Given that TB disproportionately affects socioeconomically disadvantaged populations, a more all-encompassing examination of how social determinants of health interact with AI technologies is essential to ensuring equitable and effective deployment.

A critical research gap exists in evaluating the cost-effectiveness of AI models compared to existing diagnostic methods for TB. While AI shows potential for improving diagnostic accuracy, there is insufficient analysis of its economic implications. Additionally, the need for regulatory approval and clinical validation before real-world deployment remains underexplored. Future research should focus on cost-effectiveness studies and establish clear pathways for regulatory compliance and clinical validation of AI applications in TB diagnosis and management.

Finally, ethical concerns surrounding AI in TB management also require urgent attention. Data privacy, algorithmic biases, and inequities in access to AI technologies must be addressed to ensure that AI models are effective, ethically sound, and accessible across diverse health care settings. AI models trained in high-income countries may not generalise well to LMICs, where computational resources are often limited, further exacerbating health care disparities. Exploring these ethical dimensions is instrumental to AI's equitable and responsible integration into global TB care.

## CONCLUSIONS

This scoping review highlights the potential of integrating AI-driven predictions into national TB control programmes to enhance diagnostic accuracy and facilitate the management of emerging and established pulmonary communicable diseases, and pulmonary NCDs prevalent in TB-endemic countries. By enhancing real-time surveillance, these algorithms can support sustainable health care, particularly in low-resource settings. While AI-based imaging shows promise for MDR-TB diagnostics, it should complement, rather than replace, WHO-endorsed molecular and culture-based diagnostics. Advancing AI-driven approaches in TB care could bolster targeted interventions, improve treatment outcomes, and accelerate progress toward TB elimination in vulnerable communities.

Despite its promise, AI in TB management requires further validation through large-scale prospective multicentric studies spanning diverse regions and populations. Ensuring that AI models meet the diagnostic criteria for high-risk groups and varied settings is crucial for realising their full public health impact. By advancing TB triage and enabling more targeted interventions, AI can play a pivotal role in global TB control efforts, contribute to the reduction of transmission, improve treatment success, and ultimately support the elimination of TB in vulnerable communities.

## Additional material


Online Supplementary Document
