# Supplementary material for: Artificial intelligence for tuberculosis control: a scoping review of applications in public health
Source: J Glob Health. 2025 Jul 25;15:04192. doi: 10.7189/jogh.15.04192 (PMC12290985; doi:10.7189/jogh.15.04192)
Supplement: Online Supplementary Document [file jogh-15-04192-s001.pdf]

Supplement to: Menon S, Koura KG. Artificial intelligence for tuberculosis control: a scoping review of applications in public health. J Glob Health. 2025;15:04192.

Checklist S1. Preferred Reporting Items for Systematic reviews and Meta-Analyses extension for Scoping Reviews (PRISMA-ScR) Checklist

SECTION	ITEM	PRISMA-ScR CHECKLIST ITEM	REPORTED ON PAGE #
**TITLE**			
Title	1	Identify the report as a scoping review.	1
**ABSTRACT**			
Structured summary	2	Provide a structured summary that includes (as applicable): background, objectives, eligibility criteria, sources of evidence, charting methods, results, and conclusions that relate to the review questions and objectives.	2
**INTRODUCTION**			
Rationale	3	Describe the rationale for the review in the context of what is already known. Explain why the review questions/objectives lend themselves to a scoping review approach.	4
Objectives	4	Provide an explicit statement of the questions and objectives being addressed with reference to their key elements (e.g., population or participants, concepts, and context) or other relevant key elements used to conceptualize the review questions and/or objectives.	4
**METHODS**			
Protocol and registration	5	Indicate whether a review protocol exists; state if and where it can be accessed (e.g., a Web address); and if available, provide registration information, including the registration number.	5
Eligibility criteria	6	Specify characteristics of the sources of evidence used as eligibility criteria (e.g., years considered, language, and publication status), and provide a rationale.	6
Information sources*	7	Describe all information sources in the search (e.g., databases with dates of coverage and contact with authors to identify additional sources), as well as the date the most recent search was executed.	6-13
Search	8	Present the full electronic search strategy for at least 1 database, including any limits used, such that it could be repeated.	5
Selection of sources of evidence†	9	State the process for selecting sources of evidence (i.e., screening and eligibility) included in the scoping review.	5
Data charting process‡	10	Describe the methods of charting data from the included sources of evidence (e.g., calibrated forms or forms that have been tested by the team before their use, and whether data charting was done independently or in duplicate) and any processes for obtaining and confirming data from investigators.	6
Data items	11	List and define all variables for which data were sought and any assumptions and simplifications made.	6
Critical appraisal of individual sources of evidence§	12	If done, provide a rationale for conducting a critical appraisal of included sources of evidence; describe the methods used and how this information was used in any data synthesis (if appropriate).	13-18
Synthesis of results	13	Describe the methods of handling and summarizing the data that were charted.	6

St. Michael's
Inspired Care.
Inspiring Science.

SECTION	ITEM	PRISMA-ScR CHECKLIST ITEM	REPORTED ON PAGE #
**RESULTS**			
Selection of sources of evidence	14	Give numbers of sources of evidence screened, assessed for eligibility, and included in the review, with reasons for exclusions at each stage, ideally using a flow diagram.	6-13
Characteristics of sources of evidence	15	For each source of evidence, present characteristics for which data were charted and provide the citations.	6-13
Critical appraisal within sources of evidence	16	If done, present data on critical appraisal of included sources of evidence (see item 12).	NA
Results of individual sources of evidence	17	For each included source of evidence, present the relevant data that were charted that relate to the review questions and objectives.	Table
Synthesis of results	18	Summarize and/or present the charting results as they relate to the review questions and objectives.	6-13
**DISCUSSION**			
Summary of evidence	19	Summarize the main results (including an overview of concepts, themes, and types of evidence available), link to the review questions and objectives, and consider the relevance to key groups.	13-14
Limitations	20	Discuss the limitations of the scoping review process.	15
Conclusions	21	Provide a general interpretation of the results with respect to the review questions and objectives, as well as potential implications and/or next steps.	16
**FUNDING**			
Funding	22	Describe sources of funding for the included sources of evidence, as well as sources of funding for the scoping review. Describe the role of the funders of the scoping review.	17

JBI = Joanna Briggs Institute; PRISMA-ScR = Preferred Reporting Items for Systematic reviews and Meta-Analyses extension for Scoping Reviews.

* Where *sources of evidence* (see second footnote) are compiled from, such as bibliographic databases, social media platforms, and Web sites.

† A more inclusive/heterogeneous term used to account for the different types of evidence or data sources (e.g., quantitative and/or qualitative research, expert opinion, and policy documents) that may be eligible in a scoping review as opposed to only studies. This is not to be confused with *information sources* (see first footnote).

‡ The frameworks by Arksey and O'Malley (6) and Levac and colleagues (7) and the JBI guidance (4, 5) refer to the process of data extraction in a scoping review as data charting.

§ The process of systematically examining research evidence to assess its validity, results, and relevance before using it to inform a decision. This term is used for items 12 and 19 instead of "risk of bias" (which is more applicable to systematic reviews of interventions) to include and acknowledge the various sources of evidence that may be used in a scoping review (e.g., quantitative and/or qualitative research, expert opinion, and policy document).

From: Tricco AC, Lillie E, Zarin W, O'Brien KK, Colquhoun H, Levac D, et al. PRISMA Extension for Scoping Reviews (PRISMA-ScR): Checklist and Explanation. Ann Intern Med. ;169:467–473. doi: 10.7326/M18-0850

Table S1. Characteristics of included studies

Author/Country	AI tool	Objective	Sample size	Study design/ Recruitment strategy	Population (age and exclusion or inclusion criteria)	Blinding	Results & conclusions
Okada et al. (2024) Cambodia	Artificial intelligence-based computer-aided detection (AI-CAD)	Compare AI-CAD versus Xpert and human interpretation in community-based ACF for TB in Cambodia	8,519 CXR images and medical data of participants in the ACFs	Cross-sectional study; Random selection across 32 districts	Participants (≥55 years) with any TB symptom, and persons at risk for TB	Yes	The AI-CAD system showed strong correlation with CXR classifications, in a high TB prevalence setting, achieving an AUROC of 0.86 (95% CI: 0.83–0.89) against bacteriological reference. For triage, it reduced the need for human reading and bacteriological testing by 21% and 15%, respectively, while detecting 95% of Xpert-positive TB cases. In screening, it identified 98% of Xpert-positive cases.
Alege et al. (2024) Nigeria	AI driven Bayesian predictive model for hotspot mapping	Compare AI model versus aggregated notification data to assess AI-driven hotspot mapping for TB ACF across 4 southwestern states	7,088 population clusters	Multicentric retrospective cohort analysis of ACF data (non-random selection of communities for screening)	No socio demographic data, age restriction or medical restrictions specified	Unclear/not stated	AI-driven hotspot mapping improved ACF efficiency, yielding 1.75x higher TB positivity in predicted areas
Khan et al. (2020) Pakistan	Deep learning-based chest x-ray software (CXR)(qXRv2 and CAD4TBv6)	Compare diagnostic accuracy of AI-CXR software as TB triage tests versus mycobacterial culture in a tertiary care hospital in Karachi	2,198 participants	Single centric prospective cohort study with consecutive sampling	Eligible individuals (≥15 years) and presented with TB symptoms or was a household contact of someone with active TB Median age 33(IQR: 23-49). All were HIV negative	Yes	Both software demonstrated non-inferior accuracy to WHO-recommended minimum values (qXRv2 sensitivity 0.93 (95% CI 0.89–0.95), non-inferiority p=0.0002; CAD4TBv6 sensitivity 0.93 (95% CI 0.90–0.96), p<0.0001; qXRv2 specificity 0.75 (95% CI 0.73–0.77), p<0.0001; CAD4TBv6 specificity 0.69 (95% CI 0.67–0.71), p=0.0003. Sensitivity was lower for smear-negative pulmonary TB and women (for CAD4TBv6), while specificity decreased in men, those with previous TB, older individuals, and those with lower BMI.
Nsengiyumva et al. (2021) Pakistan	AI-based CXR analysis	Compare cost-effectiveness of AI-CXR versus smear microscopy/Gene Xpert triage for TB in Karachi	2,198 participants	Single centric prospective cohort study with unclear sampling strategy	The cohort included HIV-negative individuals (≥15 years) with TB symptoms referred for testing at a TB clinic	Unclear/not stated	AI-based CXR triage lowered costs by 19% and averted 3–4% more DALYs compared to smear microscopy, and reduced costs by 37% while preventing 4% more DALYs compared to GeneXpert.
Bosman et al. (2024) Lesoto and South Africa	CAD4TBv7 (AI-based chest X-ray analysis)	Compare CAD4TBv7 versus C-reactive protein for TB triage across 10 primary health care clinics and a government hospital.	1,392 adults	Multicentric prospective cohort study with consecutive recruitment	Adults (≥18 years) presenting with ≥1 of the 4 cardinal TB symptoms were recruited. (48% with HIV)	Yes	CAD4TBv7 and CRP had AUCs of 0.87 (95% CI: 0.84–0.91) and 0.80 (95% CI: 0.76–0.84), respectively. At 90% sensitivity, specificity was 68.2% (95% CI: 65.4–71.0%) for CAD4TBv7 and 38.2% (95% CI: 35.3–41.1%) for CRP. CAD4TBv7 performed comparably to an expert radiologist and nearly met TPP criteria for TB triage.
Biewer et al. (2024) Peru	qXR v3.0 and v4.0 (AI-based chest X-ray analysis).	Compare qXR versus culture and Xpert to serve a as a triage and screening tool for TB in a tertiary-care referral hospital in Lima	578 patients (387 triage cohort, 191 screening cohort)	Cross-sectional study embedded in a larger prospective study with consecutive sampling. Random sampling for the screening cohort	Adults (≥18 years) with cough or TB risk factors, excluding those on TB treatment	Yes	qXR v4 demonstrated 91% sensitivity (95% CI: 81–97%) but low specificity (32%, 95% CI: 27–37%) for pulmonary TB diagnosis in the triage cohort, with no AUC difference from v3. In the screening cohort, specificity exceeded 90%, though only one Xpert-positive case was found. qXR detected high lung abnormality rates (81% opacities, 62%

							consolidation). While highly sensitive, its low specificity limited diagnostic value in hospitalised TB risk groups.
Luo et al (2022), China	Conditional Random Forests (cforest) model (machine learning for diagnostic algorithm)	Compare machine learning-based cforest versus forest model to distinguish active TB from LTBI using culture and/or GeneXpert MTB/RIF as references across 2 hospitals	1,155 participants (892 in discovery cohort from Tongji Hospital; 263 in validation cohort from Sino-French New City Hospital	Multicentric prospective cohort study (two cohorts: validation and discovery) with convenience sampling	Patients who received anti-TB within 1 month prior to enrolment and who were < 18 years were excluded. In the active TB discovery cohort, mean age was 52.38 ± SD 14.04 and 53.08 ± SD 14.47 in the LTBI group	Unclear/not stated	The model demonstrated AUC of 0.978 for distinguishing Active TB from LTBI, with high sensitivity (93.39%) and specificity (91.18%). The findings support the potential of machine learning in clinical diagnostics for *Mtb* infection.
Nijiati et al (2022) China	3D ResNet-50 model	Compare deep-learning-based CT image with radiologists to distinguish between Active TB (ATB) and non-ATB using CT scan images.	2,291 patients from The First People's Hospital of Kashi, China (1,160 with ATB and 1,131 with non-ATB), with an additional 200 patients (100 ATB, 100 non-ATB) from Shache County Hospital for external validation	Multicentric retrospective cohort with convenience sampling followed by random group for model development	Mean age (54.62 ± SD 17.21 years) in the active pulmonary TB group and in the non-active pulmonary TB group (45.12 ± SD18.52)	Yes	The model demonstrated superior diagnostic accuracy, outperforming radiologists, with an AUC of 0.961 in the internal testing set and 0.946 in the external testing set. Findings underscore its potential as a powerful bedside tool for TB differential diagnosis.
Liu et al (2023) Taiwan	Deep Neural Network (deep learning using chest X-rays)	Compare deep neural network with the performance of pulmonologists to distinguish between pulmonary TB, NTM-LD, and other imitators based on chest X-ray images	1,500 chest X-rays (500 from TB patients, 500 from NTM-LD patients, and 500 from imitators with negative mycobacterial cultures) collected from two hospitals	Multicentric cross sectional study with random sampling	Active pulmonary TB group (age: 54.62 ± SD 17.21 years and in the non-active pulmonary TB group (age: 45.12 ± SD 18.52 years). Patients with extensive pleural effusion were excluded	Yes	The Deep Neural Network showed satisfactory performance, with AUCs of 0.83 ± 0.005 and 0.76 ± 0.006 for pulmonary TB, 0.86 ± 0.006 and 0.64± 0.017 for NTM-LD, and 0.77± 0.007 and 0.74 ± 0.005 for the imitator group. It outperformed pulmonologists in classification accuracy and maintained stable performance across different prevalence scenarios.
Wang et al (2021) China	3D-ResNet model (deep learning framework using CT images)	Compare the 3D-ResNet deep learning model with 3 radiologists in distinguishing NTM-LD from MTB-LD using chest CT images at Tijanjin Haihe Hospital	1,105 chest CT images (301 NTM-LD and 804 MTB-LD patients) for training, validation, and testing, with an external test set of 80 patients (40 NTM-LD and 40 MTB-LD)	Multicentric retrospective cohort with random sampling	Overall mean age was 48.87 ± SD 18.32. Patients with other pulmonary diseases and who had a history of lung surgery were excluded.	Yes	The model achieved AUCs of 0.90 in training, 0.88 in validation, 0.86 in testing, and 0.78 in the external test set. The model outperformed radiologists in distinguishing NTM-LD from MTB-LD and automatically identified abnormal lung areas with over 1,000 times more efficiency, demonstrating its potential as a rapid diagnostic tool.
Yang et al (2021) China	ResUNet network model (deep learning algorithm)	Compare the performance of radiologists with and without AI assistance in distinguishing COVID-19 infected pneumonia patients from other pulmonary infections on CT scans across 3 hospitals	694 cases (118 COVID-19 pneumonia and 576 other pulmonary infections) with 111,066 CT slides, evaluated with and without AI assistance by three radiologists	Multicentric retrospective cohort with convenience sampling	Mean age of patients with TB. Overall mean age was 44 (SD ±20)	Yes	The final model achieved a test accuracy of 0.914, with an AUC of 0.903, sensitivity of 0.918, and specificity of 0.909. When used alongside radiologists, the deep learning model improved their performance in distinguishing COVID-19 from other pulmonary infections, increasing the average accuracy from 0.941 to 0.951 and sensitivity from 0.895 to 0.942.

Nabulsi et al (2021) India, China, and the US	Deep learning model (AI system)	Compare the performance of the deep learning system against multiple independent datasets, including clinical, TB, and COVID-19 cases	248,445 patients in training data from a multi-city hospital network in India; evaluated on 6 international datasets, including 2 TB and 2 COVID-19 datasets	Multicentric retrospective study with convenience sampling	No sociodemographic data, age restriction or medical restrictions specified	Yes	The AI model generalised well to new populations and unseen diseases, with AUCs varying between 0.95 (95% CI 0.93–0.97) and 0.97 (95% CI 0.94–0.99) for both TB dataset. It reduced turnaround time for abnormal cases by 7-28% in a simulated workflow, supporting its potential use for signalling abnormalities in diverse clinical settings.
Liu et al (2019) China	Random Forest model (RF) (machine learning)	Compare 3 machine learning algorithms, SVM, FNN-BP, and RF to develop a radiomics-based prediction model for differentiating silicosis and TB nodules using CT images	53 silicosis patients (139 lesions) and 89 TB patients (119 lesions)	Single centric, retrospective study. Unclear sampling	Patients who underwent routine CT scans. Mean age unclear	Unclear/not stated	The RF model demonstrated the highest accuracy (83.1%) for differentiating silicosis from tuberculosis nodules, with a sensitivity of 0.76, specificity of 0.90, and an AUC of 0.917 (95% CI: 0.8431–0.9758). RF outperformed SVM and FNN-BP, showing a significantly larger AUC (P < 0.05).
Feng et al (2020) China	Machine learning (Deep Learning Nomogram)	Compare a deep learning nomogram with individual models to differentiate between tuberculous granuloma and lung adenocarcinoma in pulmonary nodules.	550 patients (training set: 218; internal validation: 140; external validation: 192)	Multicentric retrospective cohort with convenience sampling	Mean age: Training TB cohort: 49.92 ± 13.48; Internal validation TB cohort: 53.68 ± 10.19; External TB validation cohort: 56 ± 11.36 . No medical restrictions specified	Unclear/not stated	The model incorporating deep learning features, clinical factors, and CT findings, demonstrated high diagnostic accuracy in distinguishing TBG from LAC, with AUCs of 0.889 (95% CI: 0.839-0.927), 0.879 (95 % 0.813-0.928), and 0.809 (95% CI, 0.746-0.862) in the training, internal validation, and external validation cohorts, respectively. This model can differentiate between lung adenocarcinoma and tuberculous granuloma.
Liu et al (2024) China	Machine learning (Support Vector Machine - SVM)	Compare nine machine learning algorithms for the early diagnosis of tuberculous pleural effusion (TPE)	1,435 patients, 433 with TPE and the remaining 1002 were non TPE with pleural effusions An independent cohort of 153 patients with PEs was used for external validation	Single centric retrospective cohort with convenience recruitment	Median age of non-TPE patients and TPE patients were 68.0 (18.0–97.0) and 47.0 (18.0–95.0) years, respectively. Treated TB patients were excluded	Unclear/not stated	The SVM model showed the best performance in predicting TPE, achieving an accuracy of 87.7%, an AUC of 0.914, and a sensitivity of 94.7%. The model was validated using external data.
Ren et al (2019) China	Machine learning (Random Forest - RF)	Compare machine learning algorithms for diagnosing TPE (logistic regression, k-nearest neighbors support vector machine, and random forest	192 TPE, 54 parapneumonic pleural effusion (PPE), 197 malignant pleural effusion (MPE) who underwent thoracentesis	Single centric retrospective cohort with convenience recruitment	Patients with TPE, PPE, and MPE who underwent thoracentesis and those with transudative pleural effusion were excluded. Median age with TPE 36.5(IQR:24.3-59) and 67 (IQR 56-77) with non-TPE	Unclear/not stated	The RF model outperformed other machine learning algorithms and pleural fluid adenosine deaminase, achieving a sensitivity of 90.6% and specificity of 92.3%. In a prospective study, the RF model had 100% sensitivity and 90% specificity. The RF model offers a faster and more effective approach to diagnosing TPE.
Kim et al (2024) South Korea	AI-based radiographic extent analysis	Predict TB treatment success and culture conversion using AI-analysed chest X-rays and Xpert MTB/RIF assay cycle threshold (Ct) values across 6 referral centres. (no comparison with traditional statistical methods)	230 patients with rifampicin-susceptible pulmonary TB	Multicentric retrospective study, with convenience recruitment. No comparison with traditional statistical models	Adults (age > 19) diagnosed with confirmed rifampicin-susceptible pulmonary TB. Median age: 61 (IQR:51-76)	Unclear/not stated	AI-based TB extent scores from chest X-rays were significant predictors of treatment success (OR 0.938) and culture conversion at 8 weeks (liquid medium (OR 0.91; 95 % 0.85-0.97), solid medium: (OR 0.91;95 % CI;0.85-0.97). Xpert Ct values were not significantly associated with outcomes. AI-based radiographic scoring offers potential for personalised management of pulmonary TB.

Higashiguchi et al (2021) Japan	Convolutional neural networks (CNN)	Predict the duration needed to achieve culture negativity in patients with active pulmonary TB using CNNs and chest radiographs	239 patients with culture-confirmed active pulmonary TB, divided into training (N = 180) and validation (N = 59) datasets	Multicentric retrospective cohort, with random sampling	Patients with a history of TB, or coexisting conditions that may affect chest X-ray findings, patients with extensive pleural effusion, or with NTM were excluded. No sociodemographic data	Unclear/not stated	The CNN model's predictions were significantly associated with actual culture negativity duration (Pearson's correlation coefficient = 0.392, p = 0.002), although the accuracy was limited. CNNs identified radiographic findings relevant to prediction.
Asad et al (2020) Azerbaijan, Belarus, Georgia, India, Moldova, and Romania.	Machine learning-based framework	Predict TB treatment failure using feature selection techniques and classification algorithms across 6 high TB burden countries. (No comparison with traditional statistical methods)	Multinational patient data (sample size unknown)	Multicentric retrospective analysis with convenience sampling	No sociodemographic data, age restrictions, or medical restrictions specified	Unclear/not stated	The machine learning-based framework achieved an average accuracy of 78% on the combined dataset and 92% on Romania's data. The findings underscore the importance of demographic-based features in predicting TB treatment failure.
Rodrigues et al (2024) Brazil	Machine learning-based predictive models	Predict loss to follow-up during TB treatment using national registry data from Brazil (SINAN)	243,726 cases from the Brazilian Notifiable Disease Information System. Three models (Logistic Regression, Random Forest, Light Gradient Boosting) were developed	Multicentric retrospective cohort study using data from a national database	Participants under 18 and vulnerable populations (homeless individuals, incarcerated people, pregnant women, immigrants, and healthcare workers). No mean/median age provided. HIV population (7.21%)	Unclear/not stated	41,373 experienced LTFU, and 202,353 were successfully treated. Light Gradient Boosting had the best prediction performance with an AUC between 0.71 and 0.72. A user-friendly web tool was developed to assist decision-making and improve TB treatment adherence.
Liao et al (2023) Taiwan	6 machine learning algorithms, including (1) multilayer perceptron (MLP), (2) LightGBM, (3) random forest, (4) XGBoost, (5) logistic regression, and (6) support vector machine (SVM)	Compare 6 AI algorithms to predict adverse effects (acute hepatitis, acute respiratory failure) and mortality TB treatment across 1 medical centre, 1 regional hospital, and one district hospital in Taiwan	2,248 TB patients	Multicentric retrospective cohort study with convenience sampling	Adult patients (age ≥ 20 years). Overall mean age 67.7 (SD: 16.4)	No	The models demonstrated high predictive accuracy for acute hepatitis (AUC 0.920–0.766), respiratory failure (AUC 0.884–0.797), and mortality (AUC 0.834–0.737), providing a valuable tool for early detection of adverse prognosis in TB patients.
Sethanan et al (2023) Thailand	Ensemble deep learning model embedded in a web application (TB-DRD-CXR)	Compare TB-DRD-CXR web application's ensemble deep learning model to state-of-the-art methods and standard CNN architectures using the Portal dataset (TB data science for public health impact)	The dataset included 5,039 CXR images associated with tuberculosis, comprising 1,608, 470, 2098, 108, and 755 images for DS-TB, DR-TB, MDR-TB, pre-XDR-TB, and XDR-TB, respectively	Multicentric cross sectional data with convenience sampling	No sociodemographic data, age restrictions, or medical restrictions specified	Unclear/not stated	The model outperformed existing models by 4.0%-33.9% in accuracy, achieving a 96.7% accuracy rate, making it an effective and efficient tool for MDR-TB classification.
Herman et al (2021) Indonesia	Artificial Neural Network (ANN), deployed in the	Compare performance of the ANN-based	487 participants (32 MDR, 57 Rifampicin resistant (RR) group	Multicentric prospective study with	Patients with RR /MDR regimen before DST were	Yes	The ANN-based CUHAS-ROBUST model achieved an accuracy of 88% (95% CI: 85–91) and sensitivity of 84%

	CUHAS-ROBUST application	CUHAS-ROBUST model with other AI classifiers (Logistic Regression, Decision Tree, Random Forest, Extreme Gradient Boost)	(RR-TB), 398 drug-sensitive) for model building and 157 participants (23 MDR, 21 RR) for testing	consecutive recruitment	excluded. Mean age in the RR/ MDR group: 44.06 +/- 11.57 and in the non RR group: 39.59 +/- 13.84		(95% CI: 76–89), outperforming other AI models. It showed lower specificity 90% (95% CI: 86–93) compared to Logistic Regression 99% (95% CI: 97–99). The model, integrated into the CUHAS-ROBUST application, offers a useful tool for RR-TB screening, especially in settings without GeneXpert.
Tulo et al (2022)	Computer-aided diagnostic system using machine learning techniques	Compare the diagnostic performance of machine learning classifiers using lung and mediastinum features extracted from chest X-rays to differentiate between Drug sensitive (DS)-TB, MDR-TB, and XDR-TB	CXR images obtained from a public database (TB DEPOT (data exploration portal): a multidomain tuberculosis data analysis resource) for DS and DR-TB patients	Public database, unclear recruitment	No sociodemographic data, age restrictions, or medical restrictions specified	Unclear/not stated	Incorporating mediastinal features with lung characteristics, significantly improved the diagnostic performance. The Multi-Layer Perceptron classifier achieved maximum F-measures of 82.4%, 81.0%, and 87.0% for differentiating DS vs. MDR, MDR vs. XDR, and DS vs. XDR TB, respectively, indicating clinical relevance and utility in early detection of DS and DR TB.
Portelli et al (2020)	Structure-based machine learning predictor for rifampicin resistance	Compare a computational rifampicin resistance predictor, SUSPECT-RIF (StrUctural Susceptibility PrEdiCTion for RIFampicin), to the current gold-standard GeneXpert-MTB/RIF	Use of clinical *M.tb* sequencing information training set contained 203 resistant, and 28 susceptible mutations obtained from the LSHTM. An independent test set was curated from online databases	Multicentric retrospective cohort with convenience sampling	No sociodemographic data, age restrictions, or medical restrictions specified	Yes	The predictor achieved an accuracy of up to 90.9%, with a sensitivity of 92.2%, specificity of 83.6%, and a Matthews Correlation Coefficient of 0.69, outperforming the current gold standard.
Verboven et al (2022)	Hybrid knowledge- and data-driven treatment recommender Clinical Decision Support System for DR-TB	Compare a hybrid knowledge- and data-driven Clinical decision support system for DR-TB treatment with expert recommendations	Dataset of 355 DR-TB patients with 129 unique drug resistance profiles	Proof of concept study using a multicentric retrospective cohort with convenience sampling	No sociodemographic data, age restrictions, or medical restrictions specified	Unclear/ not stated	The model achieved 95% precision. However, validation data revealed overfitting, with precision at 1 reduced to 78%. Further development and evaluation are needed in other medical fields and real-world clinical settings.
Li et al (2023) China	Radiomics-based machine learning model for predicting MDR-TB in cavitary pulmonary TB	Compare the radiomics model to the clinical and the combined model to detect MDR-TB in two hospitals	257 patients with proven active cavitary TB from two hospitals (training cohort: 187 from Beijing Chest Hospital; testing cohort: 70 from Infectious Disease Hospital of Heilongjiang Province)	Multicentric retrospective cohort with convenience sampling	No age restriction; Patients with thoracoabdominal trauma, lung cancer, silicosis, COPD, diabetes, HIV. Mean age among Drug susceptible (DS)-TB: 39.59 (SD: 15.1) and 34.87 (SD: 11.46) in the MDR TB group from the training cohort. 36.27 ± 13.12 from the DS-TB and 30.16 ± 7.49 from MDR-TB from the testing cohort	Yes	The radiomics model, using 21 features, significantly outperformed the clinical model in predicting MDR (AUC: 0.844 vs. 0.589 in the training cohort, and 0.829 vs. 0.500 in the testing cohort, $p < 0.05$), showing its strong potential as a diagnostic tool for predicting MDR in cavitary TB, improving early detection in resource-limited settings.
Yang et al (2018) United Kingdom	Machine learning models for classifying TB res	Compare machine learning models with rules set for	1839 bacterial isolates (Walker et al (2015) UK, Sierra Leone, South Africa, Germany, and	Multicentric retrospective cohort study with convenience sampling	No sociodemographic data, age restrictions, or medical restrictions specified	Unclear/not stated	The best-performing models outperformed the rules-based approach, increasing sensitivities to 97% for isoniazid, rifampicin, and ethambutol ($P < 0.01$). Sensitivities for ciprofloxacin and MDR-TB reached 96%. Moxifloxacin and

		conventional molecular diagnostics tests	Uzbekistan, representing all seven global clades				ofloxacin improved by 12% and 15%, respectively, to 95% and 96% (P < 0.01). Pyrazinamide and streptomycin sensitivities rose by 15% and 24%, to 84% and 87% (P < 0.01). The models also increased AUC by 10% for pyrazinamide and streptomycin, and 4–8% for other drugs (P < 0.01).
Sibandze et al (2020) South Africa	Machine learning to explore *M.tuberculosis (Mtb)* genotypes and disease transmission in extrapulmonary TB	Investigate *Mtb* DR and transmission patterns in extrapulmonary TB (EPTB) patients within the Tshwane metropolitan area, South Africa, using genotyping and machine learning methods. (No control group)	70 *Mtb* culture-positive non-pulmonary isolates from Extra pulmonary TB patients in Tshwane, South Africa	Multicentric retrospective cohort study with consecutive sampling	Isolates with NTB and M.bovis were excluded. The median age and range was 34 years (1-82)	Unclear/ not stated	The largest cluster comprised 25 (36%) East Asian lineage strains were significantly more associated with transmission chains compared to Euro-American and East-African Indian lineages (OR = 10.11, 95% CI: 1.56-116). EPTB forms like lymphadenitis, meningitis, and cutaneous TB were more likely linked to DR (OR = 12.69, 95% CI: 1.82-141.60).
Mohidem et al (2021) Malaysia	Artificial Neural Network (ANN)	Compare multiple Linear Regression models with ANN models in predicting the number of TB in Gombak, Selangor, Malaysia, using sociodemographic and environmental factors	Sociodemographic data of 3,325 TB, collected from the MyTB web and TB Information System database	Multicentric retrospective cohort with convenience sampling	No sociodemographic data, age restrictions, or medical restrictions specified	Unclear/not stated	Three prediction models were created using sociodemographic, environmental, and combined variables. The model incorporating both sociodemographic and environmental factors, was the most accurate with an adjusted R^2 of 0.47. The integration of sociodemographic and environmental variables resulted in more accurate TB prediction, with high accuracy above 96%.
Dixit et al (2024) South Africa	Random Forest Model	Estimate country-specific TB resistance antibiograms using *Mtb* whole-genome sequencing data and in silico resistance prediction. Validation was conducted using data from a national drug resistance survey.	*Mtb* isolates from 29 countries (n=19,149) that met sequence quality criteria	Multicentric retrospective cohort with convenience sampling	No sociodemographic data, age restrictions, or medical restrictions specified	Unclear/not stated	Validation with South Africa's national drug resistance survey showed strong alignment, with minor underestimations for isoniazid, ethionamide, and second-line injectables.
Cherniaev et al (2022) Russian Federation	Dynamic Simulation Model based on AI technologies	Compare predicted values of the epidemic situation of TB from 2017 versus actual values from 2018-2021 in Sverdlovsk oblast, Russian Federation with high levels of HIV and TB.	Statistical data on TB patients from state reporting for the period 2007-2017	Multicentric centric retrospective cohort	No sociodemographic data, age restrictions, or medical restrictions specified	Unclear/not stated	The model effectively identified and calculated trends in TB epidemiological indicators. A comparison of TB predictions from 2017 with actual data from 2018–2021 showed a strong alignment in epidemiological trends, with a maximum deviation of 14.82%.
Abade et al (2024) Brazil	Machine Learning models (SVR, XGBoost, LSTM, CNN, GRU,	Compare classical statistical methods and machine learning models for forecasting TB/HIV	Time series data analysed using classical methods to establish baseline trends and seasonality. Machine learning	Ecological time-series studies	No sociodemographic data, age restrictions, or medical restrictions specified	Unclear/not stated	Deep Learning models, particularly bidirectional LSTM and CNN-LSTM, significantly outperformed classical methods in predicting TB/HIV co-infection cases. The study highlights the effectiveness of Deep Learning approaches

	CNN-GRU, and CNN-LSTM)	co-infection cases in Mato Grosso	models captured complex dynamics and non-linearities in the data				for accurately modelling TB/HIV co-infection time series and improving forecasting accuracy.
Silva et al (2024) Brazil	Random Forest machine learning model	Compare the Random Forest model in its capacity to predict TB clusters (hot versus non-hot spots) versus the observed spatial clustering of TB cases in the riverine municipalities of the Brazilian Amazon	Data on TB incidence from 2019 to 2022 was collected from the Brazilian Health Ministry Informatics Department	Ecological time-series studies, with ML methods (Random Forest) for cluster prediction	No sociodemographic data, age restrictions, or medical restrictions specified	Unclear/not stated	The analysis revealed distinct geographical TB incidence clusters with a west-to-east distribution. The model, using 6 surveillance variables, achieved an AUC-ROC of 0.81 for predicting hot spots, using key predictors included recurrent cases, TB-related deaths, antibiotic regimen changes, new cases, and smoking history. This method may help policymakers target municipalities at high TB risk.
